# Inventory of biosecurity measures and antibiotics therapy practices on laying hen farms in Benin

**DOI:** 10.14202/vetworld.2020.2681-2690

**Published:** 2020-12-17

**Authors:** Oscar N. C. Aguidissou, Cyrille K. Boko, Camus M. Adoligbe, Clarisse H. Dete, Picole T. Capo-Chichi, Yao Akpo, Benoit G. Koutinhouin, Souaïbou Farougou

**Affiliations:** Communicable Diseases Research Unit, Applied Biology Research Laboratory, Ecole Polytechnique Abomey-Calavi, University of Abomey-Calavi, 01 BP 2009, Cotonou, Benin

**Keywords:** antibiotic therapy, bacterial diseases, biosecurity, laying hens

## Abstract

**Background and Aim:**

Laying hen breeding is on the rise in Benin; nevertheless, there are several sanitary constraints to its development, including bacterial diseases. Faced with this situation, breeders mainly resort to different means of treatment. The objective of this study was to assess the current state of hygiene measures, the bacterial diseases commonly encountered, and antibiotic therapy practices on laying hen farms in Benin.

**Materials and Methods:**

A total of 200 laying hen farms were randomly selected from lists of laying hen farms obtained from veterinary offices, territorial agricultural development agencies, and the Benin National Union of Professional Aviculturists. Each visited farmer was subjected to a semi-structured questionnaire by direct interview. The results were compared using the bilateral Z-test.

**Results:**

The results of this survey revealed that 99.5% of the surveyed farms had a health and medical prophylaxis program although only 88.5% of them reported strictly adhering to it (p<0.001). About 25.0% of them reported that the dominant bacterial diseases they commonly encountered on their farms were salmonellosis, colibacillosis, and chronic respiratory disease. Only 7.0% of farmers said that they confirmed their diagnosis outside of clinical signs through laboratory analysis. To control these pathologies, 14.5% of farmers used only oxytetracycline, while 39.0% used other antibiotics such as colistin, enrofloxacin, tylosin, tylodox, flumequine, and norfloxacin. In comparison, 13.5% used a trimethoprim-sulfadimethoxine and sulfadimidine combination, while 32.0% said that they used erythromycin, oxytetracycline, streptomycin, neomycin, and colistin (p<0.001) combination.

**Conclusion:**

This study highlights the inadequacies of hygiene and antibiotic therapy practices implemented on Benin’s laying hen farms.

## Introduction

In Africa, government policies have been developed to promote short-cycle animal husbandry ­systems, such as poultry farming, to contribute to poverty alleviation and promote food self-sufficiency [1]. Thus, poultry farming has become a very important pillar for food security and economy in many African countries, particularly in West Africa, where poultry farming is growing rapidly [2]. For example, in Benin, (a country located in intertropical zone in West Africa), poultry farming is the second-largest source of animal protein production after cattle [3] with an estimated 19,830,000 birds produced in 2017 when considering traditional breeds and a further 813,000 for modern breeds [4]. The rearing of laying hens also occupies a prominent place within the poultry sector; however, several constraints hinder its development, with bacterial diseases being a major restraint [5].

Previously, it has been shown that most poultry farmers in Benin are aware of the risk of bacterial infections, their effects on the mortality rate of laying hens, and the productivity and profitability of farms [5]. Faced with such a situation, farmers mainly resort to prevention methods such as biosecurity measures and immunization as a means of control [6,7]. However, in many cases, hygienic shortcomings result in the use of various antimicrobials in attempts to control bacterial infections and improve performances [8]. However, despite their efficacy, it is important to regulate the use of antimicrobials as their uncontrolled use is known to modify the ecology of bacteria and contribute to the selection of multidrug-resistant bacteria in animals and humans [9-13]. Another consequence of the misuse of antibiotics is the presence of active residues in animal products (eggs and meat). This can lead to adverse effects for the consumer, thus posing a public health problem [14,15]. Moreover, Boko *et al*. [16] showed that poultry farming practices in South Benin are still unsatisfactory. Given this, it is clear that urgent measures are needed that can reduce the use of antibiotics on laying hen farms. However, to develop any recommendations for farmers, knowledge of the current state of practices relating to the application of biosecurity measures and the use of antibiotics on laying hen farms is necessary.

The present study was carried out with the aim of taking stock of the biosecurity practices and bacterial diseases most commonly encountered by laying hen farmers in conjunction with their antibiotic therapy practices to be able to develop constructive recommendations.

## Materials and Methods

### Ethical approval and Informed consent

Ethical approval is not necessary for this type of study. However, informed consent was obtained from all the participants.

### Equipment

The equipment used consisted of a survey form addressed to the farmers, a global positioning system device for the recording of the geographical coordinates of the various farms visited, and a camera.

### Methods

#### Sampling plan

**Figure-1:**
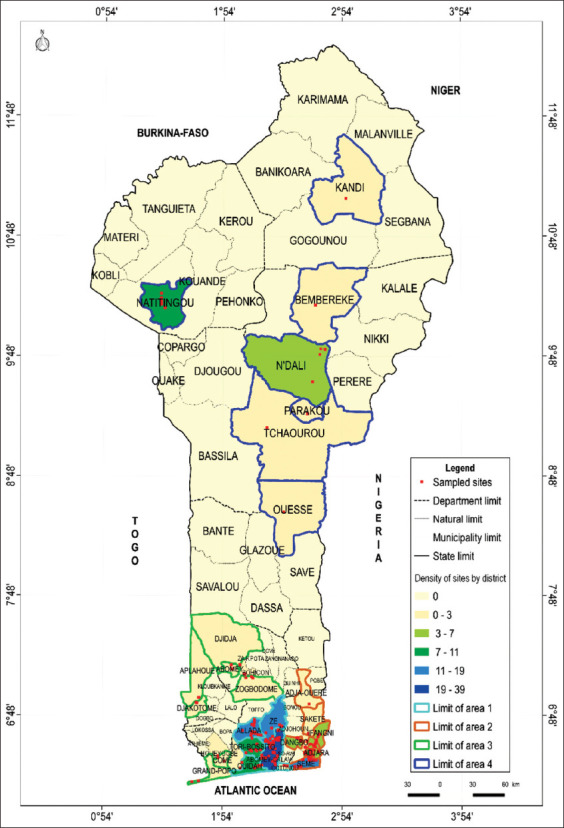
Geolocation of visited laying hen farms.

The sampling was carried out; from June to November 2019 in several municipalities (Figure-1), taking into account, the zoning adopted by the UNAP and DE in Benin and the last census carried out by the Milk and Meat Value Chain Improvement Project (PAFILAV) in 2015. In fact, in 2015, the four zones adopted by UNAP and the DE featured 719 modern poultry farmers (layers, broilers, and cockerels) with 381, 125, 168, and 45, respectively, for zone 1, zone 2, zone 3, and zone 4. The target population in this study consisted solely of laying hen breeders. Thus, 200 laying hen farms were randomly selected based on the density of farms per zone and from the lists of breeders obtained from veterinary offices, territorial agricultural development agencies (ATDA), and UNAP (Table-1). This sample size is justified by our limited resources and by the fact that some breeders refused to receive us because of the lack of feedback from the previous studies. However, according to Singleton [17], while a sample size of 2000-3000 is considered an extreme upper limit, the extreme lower limit is usually 30 cases for statistical analysis. He also adds that most social scientists would recommend a sample size of 100. Therefore, the sample size for this study is representative.

**Table-1:** Distribution of visited laying hen farms according to UNAP zoning.

Zones	Departments	Municipalities	Numbers	Percentage
Zone 1	Atlantique-Littoral	Abomey-Calavi, Zè, Allada, Cotonou,Tori-Bossito, Ouidah	103	51.50
Zone 2	Ouémé-Plateau	Sèmè, Porto-Novo, Avrankou, Ifangni, Sakété, Adjarra, Dangbo, Adja-Ouèrè	56	28.00
Zone 3	Mono-Couffo-Zou-Collines	Comè, Aplahouè, Djakotomè, Grand-Popo, Abomey, Zogbodomey, Djija	19	9.50
Zone 4	Borgou-Alibori-Atacora-Donga	Natitingou, Kandi, Tchaourou, N’dali, Ouèssè, Parakou, Bembèrèkè, Tchaourou	22	11.00
Total			200	100

### Data collection

The visited farmers were subjected to a semi-structured questionnaire by direct interview for the data collection on sociodemographic characteristics, farm management, biosecurity measures, ­dominant bacterial diseases, and antibiotic therapy practices within the farm. Each breeder was contacted 48 h before our visit for consent and to arrange an appointment. One enumerator was assigned to two zones but before the survey, the enumerators were trained on the use of the questionnaire to maintain consistency across all the interviews.

### Statistical analysis

The data collected were coded and recorded in the Excel designed database. The frequencies were calculated in relation to farm management, biosecurity measures, dominant bacterial diseases, and antibiotic treatment practices. Then, these frequencies were compared with each other using the bilateral Z-test. For each relative frequency, a 95% confidence interval (CI) was calculated according to the formula:


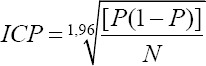


Where, P is the relative frequency and N is the sample size.

All the analyses were performed with the R 3.5.2. software (Foundation for Statistical Computing, Vienna, Australia) [18] and the graphs were designed using GraphPad Prism 5.0.0 (San Diego, California, USA) [19].

## Results

### Sociodemographic characteristics

**Figure-2:**
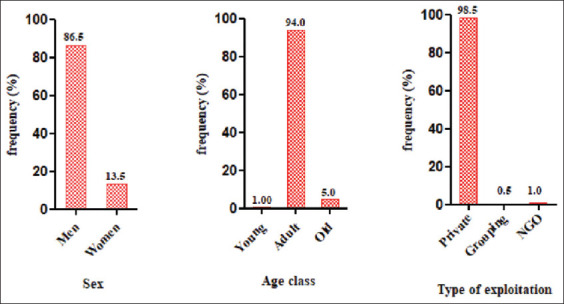
Sociodemographic characteristics of the visited farms. NGO=Non-governmental organization.

Altogether 200 farmers were selected included 103 (zone 1), 56 (zone 2), 19 (zone 3), and 22 from zone 4. Of the 200 farmers, 86.5% were male, compared to only 13.5% of females. About 94.0% were adults (25-64 years old), 5.0% were old (≥65 years old), and only 1.0% were young (21-24 years old). Almost all of the farms surveyed (98.5%) were ­privately owned (Figure-2).

### Management of the visited farms

**Figure-3:**
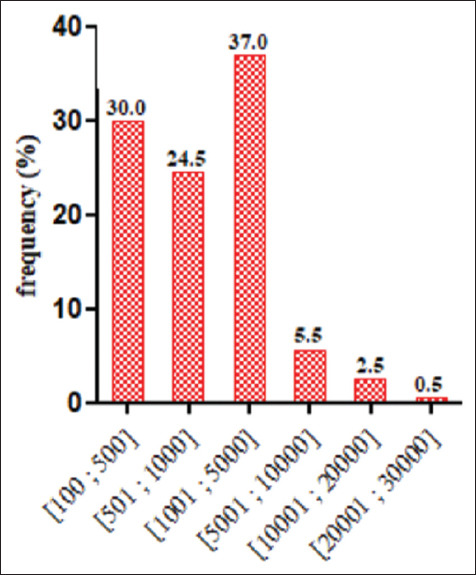
Size of livestock from visited farms.

Table-2 provides information on the management of the various farms. The table shows that 96.0% of the farmers only produced layers, 2.5% combined layers and broilers, 1.0% combined layers and cockerels, and only 0.5% produced layers, broilers, and cockerels. Although our survey revealed four breeding methods, majority of the farmers rear their animal on the ground (92.0%). On 22.5% of the farms, chickens coop was spaced 15-30 m from each other. As regards the size of the livestock on the various farms, most farmers (37.0%) owned 1001-5000 heads (Figure-3).

**Table-2:** Farms management.

Variable	Numbers	Frequency (%)	IC	Z-test
Other types of speculation				
Broilers	5	2.5^a^	2.16	***
Cockerels	2	1.0^a^	1.38	
Broilers and cockerels	1	0.5^a^	0.98	
Layers	192	96.0^b^	2.72	
Number of chicken coops				
1	57	28.5^a^	6.26	***
2-5	124	62.0^b^	6.73	
6-9	10	5.0^c^	3.02	
10 and over	9	4.5^c^	2.87	
Breeding method				
On the ground	184	92.0^a^	3.76	***
In battery	7	3.5^bc^	2.55	
Ground and battery	8	4.0^b^	2.72	
Overwater	1	0.5^c^	0.98	
Distance of 15-30 m between two chicken coops				
No	155	77.5^a^	5.79	***
Yes	45	22.5^b^	5.79	

*** =p<0.001, the frequencies of the same column followed by a different letter, differ significantly at the 1^0^/_00_ threshold

#### Biosecurity measures

**Figure-4:**
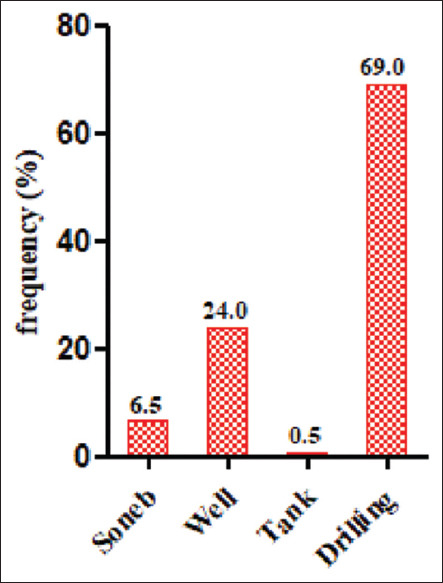
Drinking water source.

About 79.5% of the farms were fenced. Only 8.0% of these farms had wheel dips in front of their farms, while 77.0% of them had footbaths in front of each hen house. Only 12.5% of the farms stated that they had special boots for visitors. With regard to corpses management (Table-3), 80.0% of the farmers stated that they buried them, 7.5% incinerated them, and 5.0% preferred to sell them for human consumption (p<0.001). More than half (55.5%) of them said that they changed their litter when it was dirty and a minority (2.5%) did so every 6 months. About 83.5% of them said that they sold the collected litter to market gardeners, 10.5% used it in their fields near the farm. However, 1.5% of the surveyed farms stored the litter in an area on the farm. All the farmers stated that the drinking troughs are cleaned every day. With regard to the medical prophylaxis plan, (Table-3), almost all (99.5%) of the surveyed farms had a medical prophylaxis plan and 88.5% of them strictly adhered to it (p<0.001). More than half of the farmers (69.0%) fed borehole water to the animals (Figure-4) and only 18.0% of them stated that they periodically disinfected the drinking water source.

**Table-3:** Biosecurity measures.

Variable	Numbers	Frequency (%)	IC	Z-test
Presence of a fence				
Yes	159	79.5^a^	5.60	***
No	41	20.5^b^	5.60	
Presence of a gate/door				
Yes	160	80.0^a^	5.54	***
No	40	20.0^b^	5.54	
Presence of wheel dips				
Yes	16	8.0^a^	3.76	***
No	184	92.0^b^	3.76	
Presence of footbath by chicken coop				
Yes	154	77.0^a^	5.83	***
No	46	23.0^b^	5.83	
Outfits for visitors				
Yes	11	5.5^a^	3.16	***
No	189	94.5^b^	3.16	
Visitor boots				
Yes	25	12.5^a^	4.58	***
No	175	87.5^b^	4.58	
Management of corpses				
Buried	160	80.0^a^	5.54	***
Cremated	15	7.5^b^	3.65	
Thrown onto the garbage	3	1.5^c^	1.68	
Used in animal feed	12	6.0^b^	3.29	
Sold for human consumption	10	5.0^bc^	3.02	
Litter renewal frequency				
When it is dirty	111	55.5^a^	6.89	***
2 weeks	2	1.0^b^	1.38	
3 months	22	11.0^c^	4.34	
6 months	5	2.5^b^	2.16	
Every month	60	30.0^d^	6.35	
Litter management				
Composted	6	3.0^a^	2.36	***
Stored in an area on the farm	3	1.5^a^	1.68	
Sold to market gardeners	167	83.5^b^	5.14	
Spreading in nearby fields	21	10.5^c^	4.25	
Thrown onto the garbage	3	1.5^a^	1.68	
Food source				
Made by the chicken farmer	25	12.5^a^	4.58	***
Made by the chicken farmer and supplied by a factory	12	6.0^b^	3.29	
Supplied by a factory	163	81.5^c^	5.38	
Frequency of cleaning drinkers				
Every day	200	100^a^	0	***
Others	0	0.0^b^	0	
Drinking water disinfection				
Yes	36	18.0^a^	5.32	***
No	164	82.0^b^	5.32	
Medical prophylaxis plan				
Yes	199	99.5^a^	0.98	***
No	1	0.5^b^	0.98	
Compliance with the medical prophylaxis plan				
Yes	177	88.5^a^	4.42	***
No	23	11.5^b^	4.42	

*** =p<0.001, the frequencies of the same column followed by a different letter, differ significantly at the 1^0^/_00_ threshold

#### Bacterial diseases encountered and antibiotic therapy practices

About 25.0% of the farmers reported that the dominant bacterial diseases they commonly encountered were salmonellosis, colibacillosis, and chronic respiratory disease. About 18.5% reported only encountering chronic respiratory disease, 10.5% encountered both colibacillosis and chronic respiratory disease, and 25.5% encountered none of these diseases (p<0.001). Only 26.0% of the farmers stated that they were vaccinated against salmonellosis (Table-4). In the presence of these pathologies, only 49.0% of the farmers used veterinarians for diagnosis, while 39.5% did it by themselves and 11.5% used technicians. Almost half (49.0%) of the farmers said that they based their diagnosis on clinical signs, while only 7.0% of them confirmed their diagnosis by laboratory analysis outside of clinical signs (p<0.001). To treat the pathologies, 14.5% of the breeders only used oxytetracycline, 39.0% used various different antibiotics including oxytetracycline but also colistin, enrofloxacin, tylosin, tylodox, flumequine, and norfloxacin, 13.5% used a combination of trimethoprim-sulfadimethoxine and sulfadimidine, while 32.0% stated that they used a combination of erythromycin, oxytetracycline, streptomycin, neomycin, and colistin (Table-4) (p<0.001). Nearly all farmers (99.0%) said that they purchased these antibiotics from veterinary pharmacies, while 1.0% purchased them from veterinary pharmacies and the informal sector in Nigeria. In addition, more than half (56.0%) of the farmers used these antibiotics for prevention and treatment, 25.5% for treatment, and 17.5% for prevention (p<0.01). Of the 200 farmers interviewed, 74.5% of the farmers interviewed reported after treatment, they obtained the expected result (Table-4), while 4.0% of the farmers reported that they did not obtain the expected result (p<0.001).

**Table-4:** Bacterial pathologies encountered and antibiotic therapy practices.

Variable	Numbers	Frequency (%)	IC	Z-test
Dominant bacterial disease				
Salmonellosis	16	8.0^a^	3.76	***
Colibacillosis	5	2.5^b^	2.16	
Chronic respiratory diseases	37	18.5^c^	5.38	
Salmonellosis and colibacillosis	8	4.0^b^	2.72	
Salmonellosis and chronic respiratory diseases	12	6.0^ab^	3.29	
Salmonellosis, colibacillosis, chronic respiratory diseases	50	25.0^d^	6.00	
Colibacillosis and chronic respiratory diseases	21	10.5^b^	4.25	
No	51	25.5^d^	6.04	
Vaccination				
Salmonellosis	52	26.0^a^	6.08	***
No	148	74.0^b^	6.08	
Diagnostic				
Veterinary	98	49.0^a^	6.93	***
Poultry farmer	79	39.5^a^	6.78	
Technician	23	11.5^b^	4.42	
Confirmation of diagnosis				
Clinical signs	98	49.0^a^	6.92	***
Clinical signs and autopsy	74	37.0^b^	6.69	
Clinical signs, autopsy, and laboratory analysis	14	7.0^c^	3.53	
No	14	7.0^c^	3.53	
Antibiotics used				
Oxytetracycline	29	14.5^b^	4.87	***
Oxytetracycline, colistin, enrofloxacin, tylosin, tylodox, flumequine, norfloxacin	78	39.0^a^	6.75	
Trimethoprim, sulfadimethoxine, sulfadimidine	27	13.5^b^	4.73	
Erythromycin, oxytetracycline, streptomycin, neomycin, colistin	64	32.0^a^	6.46	
Prescription				
Veterinary	102	51.0^a^	6.93	***
Technician	76	38.0^b^	6.73	
Self-medication	22	11.0^c^	4.34	
Drug supply				
Veterinary pharmacy	198	99.0^a^	1.38	***
Veterinary pharmacy and market	2	1.0^b^	1.38	
How to use				
Preventive	35	17.5^a^	5.27	***
Curative	51	25.5^a^	6.04	
Preventive and curative	112	56.0^b^	6.88	
Achievement of the expected result				
Yes	149	74.5^a^	6.04	***
No	8	4.0^b^	2.72	
Often	43	21.5^c^	5.69	

*** =p<0.001, the frequencies of the same column followed by a different letter, differ significantly at the 1^0^/_00_ threshold

## Discussion

### Sociodemographic characteristics

The results of this study revealed that the majority of the laying hen breeders were male. These results are similar to those obtained in 2015 in Benin by PAFILAV [20], featuring 90.0% of men against 10.0% of women. They are also similar to those obtained by Adebowale *et al*. [11] who found that 71.8% of the interviewed farmers were male and 14.6% were female. The rearing of laying hens in Benin is, therefore, and continues to be a male-dominated activity. According to our study, this type of farming is practiced much more by adults (25-64 years old). Similar results were obtained by PAFILAV [20], who stated that 52.0% of the poultry farmers fall into the age range of 36-54 years old.

#### Management of the visited farms

In the majority of the farms, the buildings were not sufficiently spaced. These results corroborate to those obtained in Benin [16]. The proximity of livestock buildings on the farms would constitute a risk of airborne contamination from one building to another in the event of a contagious disease. Indeed, the distance between two buildings should not be less than 30 m to limit any risk of contamination [21]. On the basis of a previous study of FAO [22], poultry enterprises in Benin can be classified into three groups that include small enterprises (<1000), medium enterprises (1000-5000), and big enterprises (>5000). However, our study showed that majority of our respondents were medium-sized farm owners. Our results are different from those obtained by PAFILAV [20], who had shown that the majority of the laying hen breeders own small-sized farms. This finding could be due to the fact that breeding laying hens have developed over time [23,24] due to change in people’s lifestyle and the increasing demand in animal source food.

#### Biosecurity measures

The lack of fences in some farms could be the result of limited financial means or unwillingness of these farmers to build a fence. However, the study conducted in Mali [25] reported that many more laying hen breeders did not have a fence. This could constitute a risk of permanent exposure of the farm to live vectors which are often sources of contamination. The majority of our respondents had a footbath in front of each hen house and a few had a wheel dip. Similar observations were made by PAFILAV [20] in Benin. These results might suggest that most farmers in Benin are more aware of the importance of footbaths at the entrance to each chicken coop than of the importance of wheel dips at the entrance to the farms. Our results are different from those reported by Traoré [25], who found that only 13.04% of the farms surveyed had footbaths and none of them had wheel dips at the farm entrance. Furthermore, only 12.5% of the farmers had special boots for visitors. These results could be linked to the lack of information or negligence on the part of most farmers in managing visitors. This is because Article 6.5.5 of the Terrestrial Animal Health Code states that “All visitors entering a poultry house are required to change footwear or use a boot spray and a foot bath containing a properly maintained disinfectant” [26]. With respect to corpses, our results showed that the majority of the farmers have mastered the concept of corpses management and proceeded with burial. However, incineration remains the best method of disposal of corpses because it limits the spread and persistence of germs on farms [25]. Our results are different from those obtained by Wouembe [1], who reported that 10.0% of farmers in Cameroon bury chicken corpses. Furthermore, some of them preferred to sell the corpses for human consumption. Indeed, human consumption of dead chickens would constitute a risk factor in the transmission of certain zoonotic diseases such as salmonellosis [27]. This practice could be explained by the lack of information on zoonotic diseases among these farmers. Concerning the medical prophylaxis plan, our results corroborate those obtained in Benin [16], who also reported that 97.4% of the farms surveyed strictly applied the prophylaxis program. The failure of some farms in our study to rigorously comply with the prophylaxis program could be explained by negligence on the part of the farmers or by a lack of financial resources. The majority of producers sold the collected litter to market gardeners and a few used it in their fields close to the farm. These results are similar to those obtained by PAFILAV [20] in Benin, who also reported in their survey that 81.0% of breeders sold the collected litter to market gardeners, compared to only 16.0% who used it in fields close to their farms. This is explained by the high demand for manure by market gardeners in Benin. However, 1.5% of the farms surveyed stored the litter in one area of the farm, which exposes their farms to the risk of permanent contamination of successive strips.

#### Bacterial diseases encountered and antibiotic therapy practices

As a result of this survey, the dominant bacterial diseases commonly encountered on the farms were salmonellosis, colibacillosis, and chronic respiratory disease. The same observation was made in Algeria [28] and in Senegal [29], who also listed these diseases on laying hen farms. However, the occurrence of these diseases is most frequent in the unvaccinated farm. What may imply that failure to strictly comply with prophylactic measures by some farmers is the main reason of the occurrence of these diseases. Furthermore, the non-compliance of hygienic and sanitary rules as it is supposed to be done, the poor management of visitors and manure could also favor the persistence of these pathologies.

In the presence of these pathologies, only a handful of breeders confirmed their diagnosis outside the clinical signs by laboratory analysis. Our results are different from those obtained in Algeria [30], who reported that 25.0% of the vet monitored farms confirmed their diagnosis by laboratory analysis. Our results showed that many laying hen breeders were unaware of the importance of laboratory diagnosis and did not follow all the diagnostic steps before using antibiotics. Indeed, for an accurate diagnosis in avian pathology, after the anamnesis, a general examination should be carried out, an autopsy of a representative number of corpses should be performed and then additional laboratory tests should be requested for confirmation [25,31]. The lack of laboratory confirmation of the diagnosis by farmers could also be explained by the fact that Benin does not have enough veterinary diagnostic laboratories to assist farmers in this process. This constitutes a limitation in the strict application of the diagnostic process.

To deal with bacterial diseases, antibiotics most used for prevention and treatment by these farmers were oxytetracycline, colistin, enrofloxacin, tylosin, tylodox, flumequine, norfloxacin, erythromycin, streptomycin, neomycin, and the combination of trimethoprim-sulfadimethoxine and sulfadimidine. These results are similar to those obtained in Cameroon [32], Nigeria [11], and Benin [27]. The common use of these molecules on laying hen farms could be explained by the fact that some hatcheries and veterinary clinics offer farmers prophylactic programs in which anti-stress, anticoccidial, and antibiotic drugs are used for a long time during the growth of the animals. Moreover, failure to strictly comply with biosecurity measures and self-medication practiced by some farmers could also contribute to the excessive and uncontrolled use of these antibiotics. Furthermore, some of the farmers claimed that they did not obtain the expected result after using the antibiotics. This is likely related to ­misdiagnosis or misapplication of the prescribed antibiotics. It could also be due to the development of pathogens’ resistance to antibiotics or to the poor quality of the antibiotics, especially if they are from dubious sources. In fact, when faced with pathology on a farm, the recommended conduct generally involves the prescription of a broad-spectrum antibiotic. However, the prescription of a broad-spectrum antibiotic is applied when the offending pathogen has not yet been identified [33]. Thus, most farmers use antibiotics in an uncontrolled manner [32,34]. This practice not only promotes the presence of antibiotic residues in eggs and meat for human consumption [14,15], but also favors the emergence of multiresistant pathogens such as *Salmonella* and *Escherichia coli* on laying hen farms [5,35]. However, this resistance should not be systematically blamed in cases of treatment failures because other factors may be involved [36]. This is the case with misdiagnosis or incomplete diagnosis, a common situation when some farmers are satisfied with the clinical examination alone without resorting to laboratory tests before initiating antibiotic therapy [25]. This is also the case with the antagonistic effects associated with the combination of a bacteriostatic antibiotic with a bactericide [31].

This study thus highlights the different antibiotics commonly used by poultry breeders in Benin. It also highlights the biosecurity practices on these farms, which are still unsatisfactory, and draws attention to the importance of laboratory diagnosis by farmers. This will help to regulate or reduce the bad practices associated with antibiotic use by farmers in Benin.

## Conclusion and Recommendations

To highlight the biosecurity practices, antibiotic therapy practices, and bacterial diseases commonly encountered by laying hen breeders in Benin, a retrospective survey was carried out among breeders. At the end of this survey, it was found that most breeders make an effort to respect the hygienic-sanitary rules. However, some irregularities were observed in the management of visitors, corpses, and manure. Three major bacterial diseases such as salmonellosis, colibacillosis, and chronic respiratory disease are commonly encountered by farmers. Faced with these pathologies, they resort to several antibiotics that they use both preventively and curatively. In addition, most of them do not resort to laboratory diagnosis before the application of different antibiotics. It would, therefore, be advisable to strengthen the bodies responsible for monitoring and controlling the import and sale of antibiotic products, to increase the number of veterinary diagnostic laboratories, to raise awareness among farmers on the importance of complying with biosafety measures and laboratory diagnosis, to regulate the use of antibiotics on Benin’s laying hen farms, and finally, to promote and encourage research into pharmacological substances to limit the use of antibiotics on laying hen farms.

## Authors’ Contributions

ONCA and CKB contributed to the work designing, data collection, and manuscript drafting. CMA and CHD Clarisse performed data analysis and revised the document. PTC contributed to data collection. YA, BGK, and SF revised the document. All authors have read and approved the final manuscript.
